# The Effects of Caffeine on Exercise in Hot Environments: A Bibliometric Study

**DOI:** 10.3390/nu16213692

**Published:** 2024-10-30

**Authors:** Hansen Li, Ying Yang, Qian Liu, Liming Liu, Guodong Zhang, Xing Zhang, Mingyue Yin, Yang Cao

**Affiliations:** 1 School of Physical Education, Sichuan Agricultural University, Ya’an 625014, China; 2 Institute of Sports Science, College of Physical Education, Southwest University, Chongqing 400715, China; lygd777@swu.edu.cn; 3 Department of Physical Education and Sport, Faculty of Sport Sciences, University of Granada, 18071 Granada, Spain; 4 School of Athletic Performance, Shanghai University of Sport, Shanghai 200438, China; 5 Clinical Epidemiology and Biostatistics, School of Medical Sciences, Faculty of Medicine and Health, Örebro University, 70182 Örebro, Sweden; 6 Unit of Integrative Epidemiology, Institute of Environmental Medicine, Karolinska Institutet, 17177 Stockholm, Sweden

**Keywords:** coffee, sport, exercise, physical activity, athlete, competition, health, nutrients

## Abstract

Background: Caffeine is widely recognized as an ergogenic aid to enhance athletic performance, yet its effects in hot environments remain relatively underexplored. Aims: To provide a comprehensive overview of the research landscape and identify research themes in this field. Methods: We systematically searched the Web of Science (WoS) and SCOPUS databases using keywords related to caffeine (e.g., caffe*), hot environments (e.g., heat, hot, or therm*), and athletic performance (e.g., cardio, endurance, or strength). The Bibliometrix package in R was used for bibliometric analysis and result visualization, while a narrative review was subsequently performed to identify research themes. Results: We found that studies examining the impact of caffeine on exercise in hot conditions are relatively sparse and have progressed slowly in recent years. Research in this domain has predominantly been concentrated within an academic network led by Professor Lawrence Armstrong. Recent contributions have been sporadically made by emerging scholars, with collaborations largely confined to a few research groups and countries. Key research themes identified include exercise performance, thermoregulation, fluid balance, physiological responses, immune responses, synergistic effects with other compounds, and the influence of individual differences. Of these, the first three themes—exercise performance, thermoregulation, and fluid balance—have received the most attention. Conclusions: Caffeine’s effects on exercise performance in hot environments have not been thoroughly studied. The existing research themes are varied, and the conclusions show considerable inconsistencies. Our study highlights the need for further research into the effects of caffeine dosage, administration methods, and population-specific variables. We also call for increased collaboration among research groups to advance scientific understanding and address the gaps in this field.

## 1. Introduction

Caffeine is a natural alkaloid commonly found in various plants, with primary sources being coffee beans and tea leaves. Chemically, it is classified as a xanthine compound, with the molecular formula 1,3,7-trimethylxanthine (C_8_H_10_N_4_O_2_). Caffeine is present in numerous products such as soft drinks, energy drinks, chewing gum, and pharmaceuticals [1]. As the predominant source of caffeine, coffee consumption has increased by 67.9% over the past 26 years [2]. Between 2021 and 2022, global coffee consumption reached nearly 176 million bags (each weighing 60 kg), exceeding 10 million metric tons [3,4], highlighting the widespread global impact of caffeine.

As the most widely consumed psychoactive stimulant, caffeine can indirectly influence the release of neurotransmitters such as norepinephrine, dopamine, acetylcholine, and serotonin, potentially affecting mood, memory, alertness, and cognitive function [5]. Numerous studies have demonstrated that moderate coffee consumption can enhance cognitive function and improve mood [6,7,8].

Caffeine has a long-standing history of use in sports. Over a century ago, research first documented its positive effects on muscle function [9]. From 1984 to 2004, caffeine use was restricted in athletic competitions. However, on 1 January 2004, the World Anti-Doping Agency (WADA) removed caffeine from its list of prohibited substances and placed it under a monitoring program [10]. In recent years, a substantial body of research has consistently shown that caffeine can significantly enhance athletic performance, improving muscle endurance, exercise speed, and strength, as well as performance in both aerobic and anaerobic activities [11,12].

For anaerobic exercise, caffeine may enhance performance by stimulating the sympathetic nervous system or promoting muscle contraction signaling [13]. For aerobic exercise, caffeine’s effects are thought to stem from its ability to alter fatigue perception, reduce pain sensitivity, and change the perception of effort [13]. The ergogenic effects of caffeine are generally attributed to its role as an adenosine receptor antagonist [14]. Adenosine itself is an inhibitory neuromodulator that reduces overall neuronal activity, leading to increased fatigue and decreased alertness [15]. By antagonizing adenosine A1 and A2A receptors, caffeine enhances neural activity associated with various neurotransmitters, including dopamine, acetylcholine, norepinephrine, serotonin, glutamate, and gamma-aminobutyric acid (GABA) [15]. These mechanisms have been confirmed in animal models under strict experimental conditions, particularly with respect to the antagonism of A2A receptors [16,17].

However, the effects of caffeine can vary significantly between individuals. For instance, due to polymorphisms in the CYP1A2 isoform of cytochrome P450, the pharmacokinetics of caffeine metabolism differ among individuals [18]. Additionally, there is polymorphism at the level of another key enzyme, N-acetyltransferase 2 [18]. At the pharmacodynamic level, the primary brain target of caffeine, the adenosine A2A receptor (ADOR-A2A), also exhibits multiple polymorphisms [18]. In the context of physical performance, some researchers have highlighted that the impact of caffeine depends on factors such as dosage, timing of ingestion, and individual characteristics, including habitual caffeine consumption, training experience, gender, and genetic predisposition [19]. This underscores the complexity of caffeine’s benefits.

Exercise in hot environments presents unique challenges. Elevated ambient temperatures can limit the body’s ability to dissipate heat, increasing the risk of conditions such as muscle cramps, heat syncope, exhaustion, heat injury, and exertional heat stroke, all of which can negatively affect performance [20] In such scenarios, caffeine may reduce heat tolerance during exercise. On one hand, caffeine’s diuretic properties could exacerbate reductions in plasma volume and stroke volume. On the other hand, caffeine’s stimulation of the sympathetic nervous system might increase sweat rates. Additionally, caffeine has been shown to raise resting metabolic rates in both active and sedentary individuals, potentially increasing heat storage and body temperature [21,22].

Currently, there is limited research on the effects of caffeine on exercise performance in hot environments, and the findings remain inconsistent. Specifically, some studies have shown a significant decrease in endurance performance after caffeine consumption in hot environments compared to using a placebo, while some other studies have demonstrated a non-significant improvement [23]. Furthermore, the diversity of research objectives and varying focus on different performance metrics make it difficult to clearly understand the progress in this field. To address this gap, we conducted a bibliometric analysis and a brief review to explore the dynamics and development of this research field. This analysis is projected to offer insights for future scholars aiming to conduct new research, share findings, and seek collaborative opportunities in this field.

## 2. Methods

### 2.1. Data Sources

Web of Science (WoS) and SCOPUS are the two largest and most widely used databases, forming the foundation for many bibliometric analyses [24]. Following the methodology outlined in several bibliometric studies [25,26,27,28,29], we utilized the core databases of WoS and the complete SCOPUS database to conduct our own bibliometric analysis. Their comprehensive coverage of scientific publications ensures a robust and reliable assessment of the research landscape in this field.

### 2.2. Search Strategies

Since our focus is on the effects of caffeine on athletes or exercisers in hot environments, the study must simultaneously involve three key factors: “hot environment”, “caffeine”, and “exercise, sport, or physical activity”. In developing our search strategy, we drew on previous reviews [23,30,31]. Specifically, our search terms were defined as follows: TI = (caffe) AND TI = (hot OR heat OR temperature OR therm* OR degree) AND TI = (exercise* OR “physical activity” OR sport OR training OR workout OR fitness OR athlete* OR aerobic OR cardio OR endurance OR run* OR jog* OR bik* OR cycl* OR row* OR walk* OR hik* OR danc* OR spin* OR ski* OR sprint OR plyometric* OR resistance OR strength OR weightlift* OR bodybuild* OR powerlift* OR muscle OR yoga OR pilates OR calisthenics OR crossfit OR “martial art” OR MMA OR judo OR karate OR taekwondo OR gymnastic OR stretch* OR “tai chi” OR mobility OR stamina OR power OR flexibility OR balance OR coordination OR agility OR speed OR reaction OR explosive OR “body composition”).

The timeframe for our literature search extended from the inception of each database up to 9 September 2024. We placed no restrictions on publication type or language. Initially, 90 relevant papers were retrieved from WoS and four from SCOPUS. After screening based on titles, 35 papers were ultimately included in the bibliometric analysis.

### 2.3. Eligibility Criteria

This study focuses on the effects of caffeine on athletes or exercisers in hot environments. To achieve this, we established the following inclusion criteria.

Our eligibility criteria adhere to the Population, Intervention, Comparison, Outcomes and Study (PICOS) framework [32], as outlined below:

- P (Population): Athletes, exercisers, or any individuals capable of participating in physical activity;
- I (Intervention): The consumption of caffeine-containing substances and engagement in sports or exercise in a pre-defined hot environment;
- C (Comparison): Comparisons between caffeine use and non-caffeine, placebo, or other nutritional supplements;
- O (Outcomes): Any outcome measures related to physical performance or human health;
- S (Study design): No restrictions, including controlled trials, observational studies, and reviews.

### 2.4. Analysis

The primary objective of the presented study is to provide an overview of the field and highlight key research themes for both professional and general audiences. To achieve this, bibliometric analysis serves as a valuable and primary tool. Bibliometric analysis is a popular and rigorous method for exploring and analyzing large volumes of unstructured scientific data in fields with a substantial number of publications [33]. This approach enables researchers to uncover the nuanced evolution of specific domains while revealing emerging themes or directions [33]. Outstanding bibliometric studies can establish a solid foundation for innovation and meaningful development within a field—allowing scholars to (1) obtain a comprehensive overview, (2) identify knowledge gaps, (3) generate novel research ideas, and (4) position their expected contributions to the field [33].

We used the bibliometrix package (v. 4.1.4) in R (v. 4.3.1) for data analysis [34,35], which allows for the graphical representation of data to help visualize statistical trends.

Furthermore, due to the inherent ambiguity and lower interpretability associated with automated theme identification in bibliometric analysis, we also adopted a narrative review approach as a supplementary strategy to categorize research themes. While this method does not address quantitative research questions, it is well-suited for providing readers with the latest knowledge on specific topics or areas of interest [36].

## 3. Results

### 3.1. Bibliometric Analysis

This study included 35 publications, spanning from 1994 to 2024, and involved a total of 122 authors (Table 1). On average, each paper was cited approximately 13 times.

Many bibliometric analyses can be based on the titles of studies. Therefore, we included all relevant studies with confirmed titles in the analysis. Nevertheless, we also presented basic characteristics of studies that could be accessed in full text (Table 2). As shown in the table below, the participants in these studies varied, including trained athletes and general university students. The primary form of exercise used was cycling, although some studies also utilized walking or running [37,38,39,40,41,42,43,44,45]. In addition to using only a placebo to investigate the effects of caffeine, some studies employed combined supplements [40,46,47].

**Figure 1:**
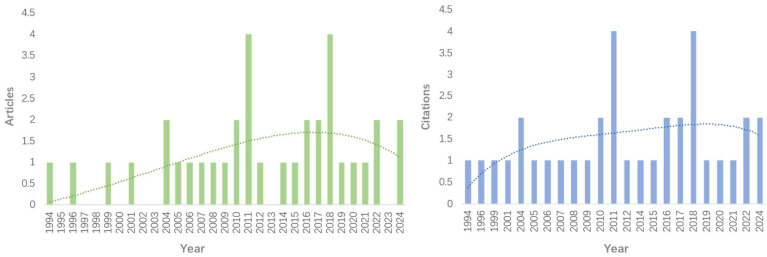
Annual trends in publication and citation counts.

The number of papers published in this field showed an upward trend over time before declining, reaching its peak between 2016 and 2018, and then experiencing a decline. Citation activity followed a similar pattern, with the majority of citations occurring during the same period (Figure 1). In other years, both publication and citation counts hovered around one paper/citation per year.

Table 3 presents the ten most frequently cited papers included in our review, with total citations ranging from 22 to 83 as of the time of our search, and average annual citations between 1.57 and 4.61. The most cited paper is authored by Armstrong, titled “Caffeine, Fluid-Electrolyte Balance, Temperature Regulation, and Exercise-Heat Tolerance”.

**Figure 2:**
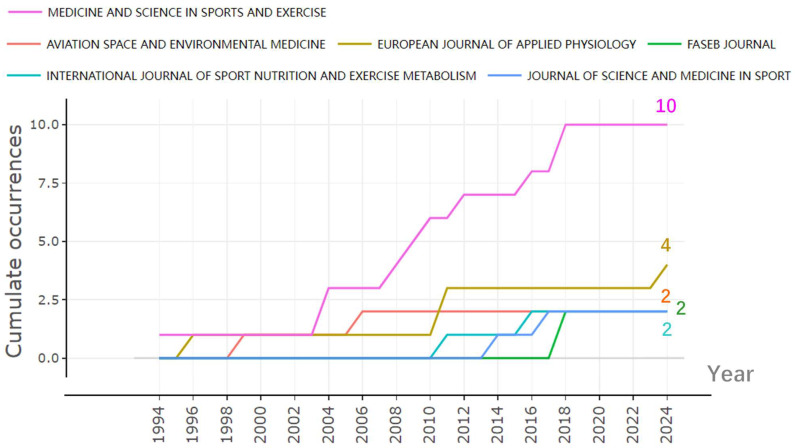
Yearly publication trends of major journal.

Figure 2 demonstrates the primary journals that published the included studies and their publication dynamics over time. *Medicine & Science in Sports & Exercise* emerged as the most prolific journal in this area, with the highest number of publications, and its output has steadily increased over time. The *European Journal of Applied Physiology* ranked second in terms of publication volume, though it only contributed a total of four papers to the field.

**Figure 3:**
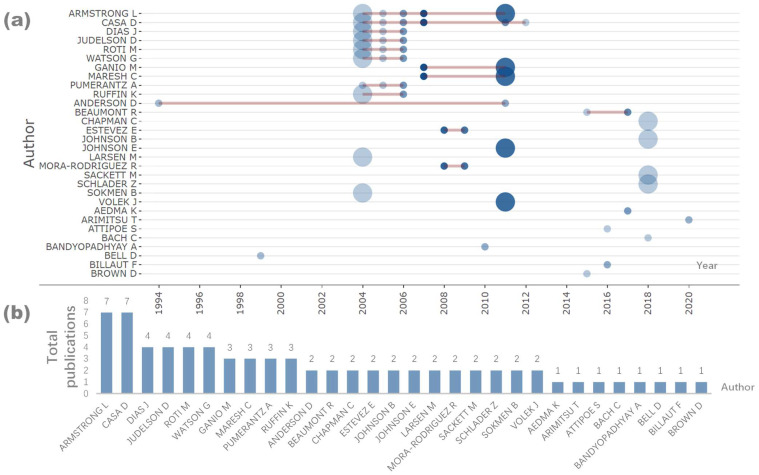
Main authors and their active periods. Note: the size of the circles represents the number of publications, with larger circles indicating a higher volume of work; the brown horizontal lines denote the time periods during which each author was actively publishing.

Figure 3 illustrates the top 30 authors in this field. Lawrence Armstrong and Douglas Casa have the highest number of publications, followed by Joao C. Dias, Daniel A. Judelson, Melissa W. Roti, and Greig Watson. Notably, these prominent authors have largely ceased their active contributions in this field since 2012.

**Figure 4:**
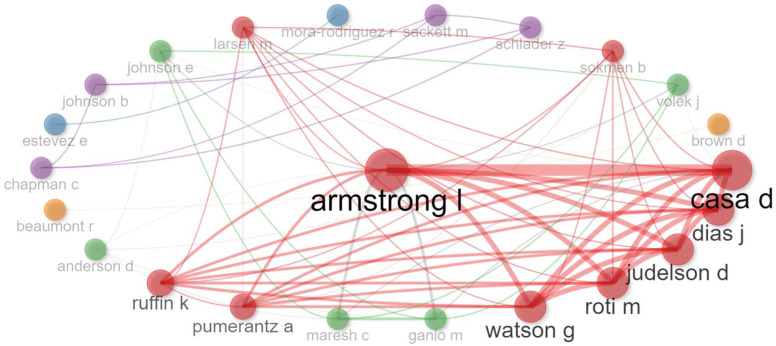
Collaboration network among 30 main authors. Note: different colors represent distinct collaboration networks; thicker lines indicate higher frequencies of co-authorship in publications.

We also examined the collaboration network among these 30 leading authors. Figure 4 reveals that most collaborative efforts are centered around Lawrence Armstrong, forming a major cluster. In contrast, collaboration among other authors is relatively sparse and confined to a few pairs.

**Figure 5:**
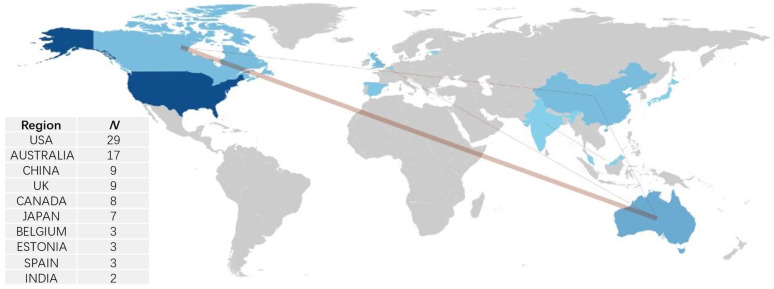
Publication Counts and Collaboration Patterns by Country/Region. Note: darker blue shades represent higher publication volumes; brown lines indicate collaboration, with thicker lines denoting higher frequencies of collaborative efforts.

Figure 5 demonstrates that the research field involves a limited number of countries or regions. The United States is the leading contributor, followed by Australia and China. Collaborative efforts are most prominent between Canada and Australia, while interactions among other countries are comparatively minimal.

**Figure 6:**
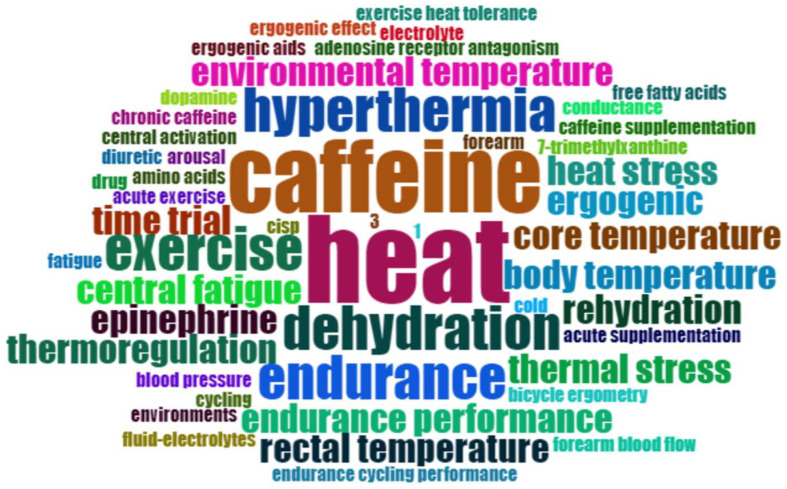
Word cloud based on authors’ keywords.

We generated a word cloud (Figure 6) based on the keywords provided by the authors of each paper. The most frequently occurring terms are “caffeine” and “heat”, which appear as the central themes. Additionally, terms such as “dehydration”, “endurance”, and “hyperthermia” also appear frequently, reflecting the core outcome measures that researchers focus on.

**Figure 7:**
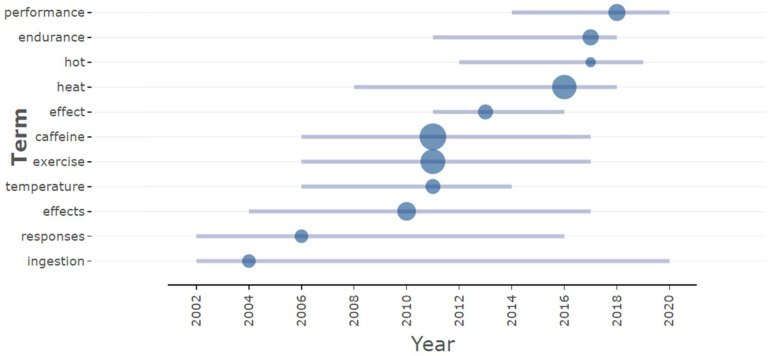
Topic trends based on titles. Note: the size of the circles represents the number of publications, with larger circles indicating a higher volume of work; blue horizontal lines denote the time span during which each theme has been prominent.

We analyzed the themes of these studies and their evolution over time based on the titles of each paper. As shown in Figure 7, terms such as “performance” (referring to athletic performance) and “endurance” (pertaining to endurance exercise) are more contemporary themes. Other terms are primarily associated with general research settings within the field.

We conducted a thematic analysis based on the keywords provided by all authors. Each theme is evaluated using two parameters: “density” and “centrality”. The position of each theme within the four quadrants is determined by these parameters, indicating the similarity of items within and between themes. The similarity of items is calculated based on the co-occurrence frequency of keywords.

**Figure 8:**
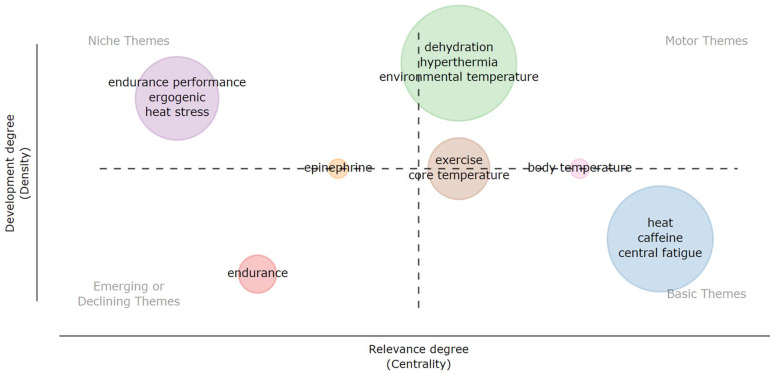
Theme map based on abstracts.

Density refers to Callon’s density [66], which measures the strength of interactions between keywords within a theme. In contrast, centrality refers to Callon’s centrality [66], which assesses the strength of interactions between keywords in a theme and those in other themes. Thus, centrality reflects the importance of a theme in the overall development of the research field, while density indicates the development stage of the theme [67]. The thematic map is divided into four quadrants (Figure 8) as follows:

(1) Upper Right Quadrant: Motor Themes—These are mature themes that are crucial to the structure of the research field, characterized by high centrality and density.
(2) Lower Right Quadrant: Basic Themes—These clusters are linked by numerous keywords but exhibit significant variability between them. They represent emerging or past themes with potential within the discipline.
(3) Upper Left Quadrant: Niche Themes—These are mature but very specialized themes, playing a minor role in the broader field.
(4) Lower Left Quadrant: Emerging or Declining Themes—These themes have the potential to evolve toward greater centrality or density. They could signify new trends or developments in the field [68].

In our thematic map, dehydration and hyperthermia are identified as mature and critical themes, while endurance is positioned as a less central theme (in the left quadrant).

### 3.2. Theme Analysis

**Figure 9:**
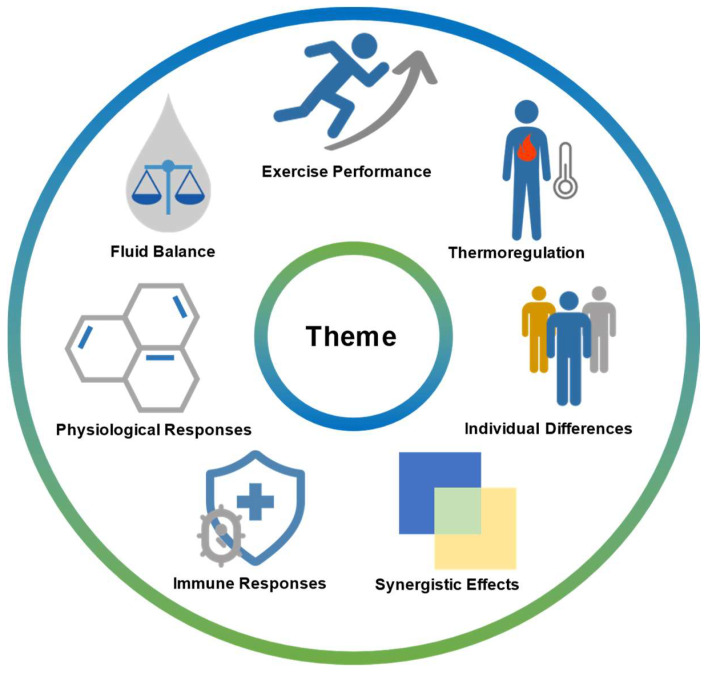
Topics of the included studies.

Given the inherent ambiguity in thematic exploration provided by bibliometric analysis, we also employed a narrative review method to summarize the main themes identified in the bibliometric analysis and organize the evidence. The primary themes can be broadly categorized as follows (Figure 9):

#### 3.2.1. The Role of Caffeine on Exercise Performance

Numerous studies have explored the effects of caffeine on exercise performance in hot environments, particularly focusing on endurance activities. For instance, Nakamura, et al. [51] found that a 3 mg/kg dose of caffeine significantly increased the total work performed by male soccer players during 90 min of intermittent sprint cycling at 32 °C and 70% humidity. Similarly, Beaumont and James [54] observed that a 6 mg/kg dose of caffeine improved the total work done during 60 min of cycling at 30 °C and 50% humidity.

Beyond single temperature settings, Ganio, et al. [58] reported that a 3 mg/kg caffeine dose enhanced total work in male cyclists during 90 min of continuous cycling at 12 °C and 33 °C (average intensity of 65% VO_2_max). Time-trial tests also indicated that a 3 mg/kg caffeine dose led to faster cycling times at 35 °C and 25% humidity.

In other exercise forms, Ping, et al. [41] found that caffeine ingestion (5 mg/kg) extended time to exhaustion for male recreational runners on a treadmill in a temperature-controlled environment (31 °C and 70% relative humidity).

To examine the effects of dosage, Beaumont, et al. [55] investigated both low-dose (3 mg/kg) and high-dose (6–9 mg/kg) caffeine, finding that low-dose caffeine improved total work in a 30-min time trial, whereas high-dose caffeine showed no difference compared to placebo.

In addition to caffeine alone, some studies have considered the role of electrolytes. Coso, et al. [63] tested endurance-trained cyclists in a 36 °C dry heat environment for 120 min (63% VO_2_max), comparing groups with no fluid intake, electrolyte intake, and caffeine plus electrolyte intake. They found that caffeine plus electrolyte intake enhanced maximal cycling power.

However, not all research provides positive evidence. For example, Roelands, et al. [60] found that trained male cyclists did not experience improved cycling performance at 30 °C with a 6 mg/kg caffeine dose. Similarly, Cohen, et al. [45] reported no improvement in performance for seven endurance-trained road cyclists (five men and two women, ages 23 to 51) after consuming 0, 5, or 9 mg/kg of caffeine in a 21 km time trial. Additionally, John, et al. [48] found no change in fatigue time for males consuming 5 mg/kg caffeine during exhaustive cycling at 35 °C and 40% humidity.

Aside from just cycling, Hanson, et al. [37] also observed that neither 3 mg/kg (low dose) nor 6 mg/kg (moderate dose) of caffeine improved 10 km run speeds.

In addition to controlled trials, a meta-analysis encompassing six studies also indicated that caffeine intake did not substantially enhance endurance performance (only non-significant improvements were observed).

In summary, the impact of caffeine on exercise performance remains unclear and may be influenced by factors such as dosage, specific environmental conditions, and the nature of the exercise.

#### 3.2.2. The Role of Caffeine on Thermoregulation

Thermoregulation is another crucial aspect, especially regarding core body temperature, heat stress responses, and thermal balance during exercise in hot conditions.

Some studies suggest that caffeine may increase thermal load. In the previous section on “Exercise Performance”, some research not only found that caffeine did not benefit exercise performance but also increased thermal burden. For example, Roelands, et al. [60] observed that caffeine did not improve cycling performance but significantly increased rectal temperature. Similarly, John, et al. [48] found that caffeine ingestion did not alter fatigue time but increased core temperature and decreased heat comfort. Hanson, et al. [37] also found that endurance runners consuming 6 mg/kg (moderate dose) of caffeine experienced a faster rise in core temperature during running in a hot environment.

On the other hand, more studies indicate that caffeine does not significantly impact thermoregulation. In the studies suggesting caffeine improves exercise performance, caffeine did not notably increase participants’ body temperature [41,51,54,56,58].

Other studies, focusing specifically on thermoregulation, also provide similar evidence. Ely, et al. [57] found that high doses of caffeine (9 mg/kg) did not lead to significant heat storage, dry heat gain, or changes in body temperature in males unaccustomed to caffeine and heat during moderate-intensity cycling in high temperatures (40 °C and 25% humidity). Another study on cycling, involving 40 min of cycling at 65% VO_2_max, indicated that while caffeine increased heart rate five minutes after exercise, it did not show a significant negative impact on temperature regulation [52]. In another similar study on cycling, Stebbins, et al. [64] hypothesized that caffeine might reduce skin blood flow in hot conditions, thereby inhibiting heat dissipation and raising body temperature. However, they did not observe changes in skin blood flow in their experiment, suggesting limited impact of caffeine.

In addition to caffeine alone, Del Coso, et al. [62] tested six different hydration strategies (no fluid, water, carbohydrate-electrolyte solution, and combinations with caffeine). The results showed that caffeine did not significantly affect heat production or heat dissipation during 120 min of cycling in a dry heat environment (63% VO_2_max). However, when combined with an electrolyte solution, caffeine slightly increased core temperature, significantly increased sweat electrolyte loss and urine output, but did not lead to dehydration or blood electrolyte imbalances.

More than just cycling, a walking test (5.6 km/h, 5% incline; dry bulb temperature, 37.7 ± 0.1 °C; relative humidity, 56.3 ± 1.5%) with long-term caffeine ingestion (3 and 6 mg/kg/day) did not alter exercise-heat tolerance, hydration status, or subjective responses after acute caffeine ingestion [42].

In summary, the impact of caffeine on thermoregulation remains contentious and requires further investigation.

#### 3.2.3. The Role of Caffeine on Fluid Balance

Several studies have investigated the effects of caffeine ingestion on fluid balance, electrolyte loss, and dehydration under high-temperature conditions.

For instance, Chapman, et al. [53] had healthy participants perform four 1-h work-rest cycles (45 min of exercise followed by 15 min of rest) in a 35 °C, 65% relative humidity environment, consuming either a caffeinated soft drink or water. The study showed no significant differences in core temperature, body weight changes, or urine specific gravity between the two groups. However, the group consuming caffeinated soft drinks experienced a significant increase in plasma osmolality, a decrease in plasma volume, and a more pronounced rise in heart rate and mean arterial pressure. These results suggest that consuming caffeinated soft drinks during exercise in hot conditions may exacerbate dehydration and increase cardiovascular strain.

In addition to acute intake, a study involving 59 male college students randomly assigned to three groups also assessed the longer-term impact of caffeine on hydration status. Participants ingested 3 mg/kg/day of caffeine for 6 days, followed by either 0, 3, or 6 mg/kg/day for another 6 days. A 90-min high-temperature walking test revealed that long-term caffeine consumption did not have a significant impact on hydration status [43,44].

To investigate post-exercise hydration, six male participants exercised for one hour in a hot, humid environment (86 ± 3 °F and 44 ± 8% relative humidity) to induce dehydration [61]. Over the 24 h following exercise-induced dehydration, the subjects participated in three trials to drink either water, caffeine-free Diet Coke^®^, or regular Diet Coke^®^ at their discretion. The results indicated that both caffeinated and caffeine-free beverages were as effective as water in restoring fluid balance. Therefore, these beverages can be used for rehydration following exercise-induced dehydration.

In summary, only a few studies suggest that short-term caffeine ingestion may exacerbate dehydration and cardiovascular strain during exercise in hot conditions. However, long-term consumption appears to have a minimal impact on overall hydration status.

#### 3.2.4. The Role of Caffeine on Physiological Responses

Several studies have explored the effects of caffeine on various physiological responses, including cardiovascular and respiratory systems, metabolic reactions, muscle pain, and kidney health.

For instance, Anderson and Hickminey [65] investigated the effects of caffeine on metabolism and catecholamine responses after mild exercise in both cold and warm environments. They found that, after ingesting 5 mg/kg of caffeine and cycling for 60 min in a warm environment (28 °C and 50% relative humidity), plasma adrenaline levels increased. However, there were no significant changes in norepinephrine levels or metabolic markers.

In another study focused on pain perception, Ganio, et al. [59] reported that caffeine ingestion (3 mg/kg) significantly reduced leg muscle pain in a hot environment (33 °C) compared to a cool environment (12 °C). Despite this, caffeine did not affect overall perceived exertion (O-RPE), local perceived exertion (L-RPE), or central perceived exertion (C-RPE).

Regarding organ damage, Chapman, et al. [69] conducted a study with 12 healthy adults who exercised in a 35 °C, 65% relative humidity environment and consumed either caffeinated or non-caffeinated soda during rest. The results indicated that the group consuming caffeinated soda had significantly higher plasma osmolality, serum creatinine, and uric acid levels after exercise. Additionally, their nighttime urine flow rate was reduced, and the incidence of acute kidney injury (AKI) was higher (75% vs. 8%). Although these markers returned to normal within 24 h, the increase in urinary NGAL levels in the caffeinated soda group suggested exacerbated kidney function impairment. These findings suggest that caffeinated soft drinks can worsen kidney function and acute kidney injury under high-temperature conditions.

In summary, caffeine’s effects on physiological responses can vary, with some studies indicating changes in hormone levels, pain perception, and kidney function, particularly under high-temperature and exercise conditions.

#### 3.2.5. The Role of Caffeine on Immune Responses

We identified only one study that specifically examined the effects of caffeine intake on the immune system. In this study, Cheng, et al. [49] conducted a double-blind, randomized crossover experiment to investigate how caffeine (6 mg/kg body weight) affects salivary antimicrobial proteins during exercise in a hot environment. Twelve endurance-trained males participated in 40 min of cycling in a 33 °C environment, with participants ingesting either caffeine or a placebo. Saliva samples were collected to measure salivary α-amylase (sAA) and lactoferrin (sLac), and core body temperature, heart rate, and rating of perceived exertion (RPE) were also monitored. Cheng, et al. [49] found that while caffeine did not significantly affect core body temperature or heart rate, it did lower perceived exertion during exercise. Additionally, caffeine significantly increased sAA activity, suggesting a potential enhancement in salivary biomarkers related to immune response. This implies that caffeine may have a beneficial effect on certain aspects of the immune system during exercise in hot conditions, though further research is needed to confirm these findings.

#### 3.2.6. Synergistic Effects of Caffeine with Other Compounds

Several studies have examined the synergistic effects of caffeine when combined with other substances, such as amino acids, taurine, or ephedrine, on exercise performance and physiological responses.

For example, Yu, et al. [46] conducted a study among university students that tested the effects of taurine, caffeine, and their combination on time to exhaustion (TTE) under high heat and humidity conditions (35 °C and 65% relative humidity). The results indicated that both taurine and caffeine supplementation improved TTE, with taurine showing the most pronounced effect. However, the combination of taurine and caffeine did not produce any additional benefit beyond taurine alone.

Similarly, Eaton, et al. [40] explored the combined effects of essential amino acids (EAA) and caffeine on reducing central nervous system (CNS) fatigue during repeated sprint running in extreme heat. Their findings suggested that the co-ingestion of caffeine and EAA was more effective in maintaining muscle activity and central drive, and it slightly improved running performance compared to caffeine or EAA alone.

In another study, Bell, et al. [47] evaluated the effects of combining ephedrine and caffeine on metabolism during moderate exercise in a hot, dry environment. Their results showed a slight increase in metabolic rate after consuming both substances, but the increased heat loss mechanisms prevented any significant rise in core body temperature, suggesting that the combination did not negatively impact thermoregulation during moderate exercise in the heat.

These studies highlight that while caffeine can enhance performance, its combination with other compounds does not always lead to an additive effect and may depend on the specific context or compound.

#### 3.2.7. Influence of Individual Differences on Caffeine’s Effects

Several studies have investigated how individual differences, such as gender, menstrual cycle, and habitual caffeine consumption, can affect the impact of caffeine on exercise performance and physiological responses.

A study focusing on gender differences found that caffeine significantly affected perceived exertion and fatigue in men, but not in women, after completing two constant-load walking sessions in a hot, dry environment (42 °C and 20% humidity) [39]. This suggests that the physiological and perceptual responses to caffeine may vary between men and women, particularly in extreme conditions.

Another study examined the effects of the menstrual cycle on caffeine’s efficacy. Rutherford and Palmer [38] aimed to evaluate how different phases of the menstrual cycle and caffeine supplementation influenced both physical and cognitive performance in women exercising in the heat. Recreationally active women using monophasic oral contraceptives completed a 5-km time trial during the early follicular (EF, days 3–5) and mid-follicular (MF, days 19–21) phases of their menstrual cycle (29.6 °C and 55.8% humidity). The results indicated that despite an increase in post-exercise oral temperature during the EF phase, caffeine improved time-trial performance in the heat, suggesting the potential benefit of caffeine regardless of menstrual cycle phase.

Regarding habitual caffeine consumption, Hunt, et al. [50] studied participants with and without a regular caffeine habit, who performed 60 min of cycling (7 W/kg) at a fixed metabolic heat production in a warm environment (30.6 °C and 31% humidity). Participants consumed either 5 mg/kg caffeine or a placebo before the trial. The findings showed that only habitual caffeine users (HAB) experienced a significantly greater increase in esophageal temperature compared to the placebo after exercise. Furthermore, the HAB group had a reduced increase in maximal skin blood flow percentage in the forearm and back with caffeine consumption, while no such effect was observed in the non-habitual group (NHAB). This highlights that caffeine’s thermoregulatory and cardiovascular effects may differ based on habitual caffeine intake.

These studies underscore that individual factors such as gender, menstrual cycle, and caffeine habits can influence how caffeine affects the body, especially under heat stress.

## 4. Discussion

We performed a bibliometric analysis and a narrative review for the topic of “Effects of Caffeine on Athletes in Hot Environments”, providing an overview of the field’s development and current state.

### 4.1. Current Research Status and Characteristics

In terms of publication volume, research in this area has consistently remained limited, with a slight decline in recent years. Caffeine and exercise studies, particularly in the context of heat, have not attracted much attention historically. Supporting this, a bibliometric analysis on caffeine and exercise between 1938 and August 2021 found only 160 relevant records in the Web of Science database [70]. Although this number is small, the ongoing presence of publications indicates that a segment of researchers continues to explore this topic. Similar trends are reported in studies on other related topics. Gutiérrez-Hellín, et al. [71] utilized bibliometric methods to investigate research trends on the effect of caffeine on fat oxidation. They found that only 182 papers were published between 1992 and 2022, showing a pattern of growth followed by a decline in recent years. Like our findings, Gutiérrez-Hellín, et al. [71] observed a recent downturn in publications, suggesting that interest in caffeine’s effects in hot environments might be waning. This declining trend in publication volume could indicate a gradual decrease in academic attention towards the effects of caffeine on exercise performance in hot conditions. However, despite the relatively small number of studies, the continuous research efforts suggest that there remains some level of ongoing exploration in this niche field.

In terms of leading journals for research on caffeine’s effects in hot environments, we found that *Medicine & Science in Sports & Exercise* is the most prominent publication, followed by the *European Journal of Applied Physiology*. This aligns closely with trends in the broader field of caffeine and exercise. Contreras-Barraza, et al. [70] similarly identified *Journal of Strength Conditioning Research* and *Medicine & Science in Sports & Exercise* as the primary journals publishing research in this area. Our trend analysis further highlights the increasing number of relevant publications in *Medicine & Science in Sports & Exercise* in recent years, reflecting growing research interest in this specific domain. The journal typically features original investigations, clinical studies, and comprehensive reviews on current topics in sports medicine and exercise science, making it a key platform for disseminating knowledge to exercise physiologists, physical therapists, team physicians, and athletic trainers. For future researchers aiming to contribute to this field, *Medicine & Science in Sports & Exercise* stands out as a valuable publication venue to consider for sharing findings and engaging with a specialized audience interested in the intersection of caffeine, exercise, and thermal stress.

In terms of influential authors, our findings highlight that Professor Lawrence Armstrong and Professor Douglas Casa are the most prolific contributors to research on the ergogenic effects of caffeine in hot environments. Both scholars have extensive experience in this area, though our analysis indicates that their focus has shifted away from related research in recent years. Lawrence Armstrong, an expert in exercise, dehydration, and metabolism, has retired but remains active in academia through collaborations. Notably, he co-authored a study on endurance exercise in hot and humid environments in 2023 [72]. Similarly, Professor Douglas Casa, known for his expertise in sports medicine and exercise physiology, published work on exercise in hot and cold environments as recently as 2022 [73]. More recently active in this field are researchers like Kevin John and Peiqi Yu, both of whom published experimental studies in 2024 [46,48]. This suggests a shift in leadership in caffeine-related research toward emerging scholars. Our analysis of collaboration networks reveals that, aside from Lawrence Armstrong’s collaborations, research partnerships in this field are sporadic and limited. At the regional level, most of the research is concentrated in a few countries, primarily the United States, Australia, and China. Although collaboration between the U.S. and Australia appears relatively strong, international cooperation beyond these regions is limited. This lack of global collaboration may hinder more in-depth research in the field. Given these findings, we encourage scholars interested in the caffeine and thermal environments to seek international partnerships.

### 4.2. Major Research Themes

One of the key focuses of this study is the thematic exploration of research on caffeine’s effects in hot environments. Our bibliometric analysis shows that between 2012 and 2020, topics related to exercise performance and endurance dominated the field. Consistently, our narrative review also identified exercise performance as the most heavily studied topic, with endurance sports, such as cycling and long-distance running, being the primary focus. Over the past decades, the relationship between caffeine and exercise performance has been widely documented [30,31,74,75,76]. However, as Naulleau, et al. [23] noted, when exercise is restricted to hot environments, the body of research becomes much smaller. In the meta-analysis by Naulleau, et al. [23], only six studies were deemed suitable for inclusion in the section on exercise performance in hot conditions, and the improvements in performance were not statistically significant. Our review of the literature also reveals inconsistencies in findings regarding exercise performance in hot environments. This variation in outcomes makes it difficult to draw definitive conclusions about caffeine’s performance-enhancing effects under thermal stress.

In addition to exercise performance, our review highlights thermoregulation as another critical theme in the research on caffeine in hot environments. This connection is understandable, as caffeine is known to have thermogenic effects [77], potentially reducing the body’s ability to adapt to heat. Alongside thermoregulation, fluid balance is a major concern for athletes. While there is a common belief that caffeine increases the risk of dehydration, evidence supporting this claim remains insufficient [21]. A recent review suggests that the diuretic effect of moderate caffeine intake during exercise may be limited, with factors such as sweat rate, hydration strategies, and genetics playing a more significant role in maintaining fluid balance [78]. In our review, only a few studies pointed to potential adverse effects of caffeine on fluid balance, reinforcing the need for further research. Beyond these primary themes, our study identified several other topics, such as the physiological responses to caffeine, individual differences in caffeine’s effects, synergistic effects with other compounds, and immune response. These areas, however, have received relatively little attention in the context of exercise in hot environments. For example, individual differences—such as habitual caffeine use and gender—have been widely discussed in other contexts [19,79], but research on these factors in hot environments is scarce and varied in design and focus, making it difficult to draw clear conclusions. The same applies to the other three themes: studies exploring the physiological, synergistic, and immune responses to caffeine in hot environments are limited. In other words, significant gaps remain in our understanding of caffeine’s impact on exercise and physical performance in hot conditions. Considering the increasing prevalence of activities and competitions in hot environments, such as marathons, we call for more focused research in this area to expand the body of knowledge and provide clearer guidance for athletes and practitioners.

### 4.3. Mechanisms of Caffeine’s Impact on Exercise or Sports

Lastly, we briefly revisit the mechanisms through which caffeine may affect exercise performance. As mentioned in the introduction, numerous studies have documented the positive effects of caffeine on athletic performance, and even on specific skills, under normal temperature conditions. Currently, the beneficial effects of caffeine on athletes or exercisers are thought to involve four primary mechanisms [80].

The first mechanism is the promotion of fat oxidation [81]. A meta-analysis indicated that acute intake of moderate doses of caffeine, following at least 5 h of fasting, could be an effective strategy for increasing fat oxidation during submaximal aerobic exercise [82]. A recent study further demonstrated that combining acute caffeine intake with dietary consumption within 5 h prior to exercise can enhance fat oxidation during submaximal aerobic activity [83]. Animal models have shown that caffeine intake can stimulate fat breakdown either by stimulating catecholamines or by antagonizing A1 adenosine receptors on fat cells [84,85], suggesting the underlying mechanism by which caffeine acts as a fat-mobilizing agent.

The second mechanism is the enhancement of exogenous carbohydrate oxidation. Yeo, et al. [86] found that co-ingestion of caffeine with glucose increased exogenous carbohydrate (CHO) oxidation compared to glucose alone, possibly due to enhanced intestinal absorption.

The third mechanism is caffeine’s positive effect on the nervous system. For instance, caffeine may influence dopaminergic neurotransmission by inhibiting adenosine receptors [81]. In animal models, caffeine has been shown to delay fatigue through central nervous system mechanisms, partly by blocking adenosine receptors [82]. Moreover, higher concentrations of caffeine might affect neurotransmission by activating calcium channels in the endoplasmic and sarcoplasmic reticula [83].

The final mechanism is the placebo effect of caffeine [87], which is rarely studied but comprehensible.

In conclusion, these mechanisms may help explain the potential benefits of caffeine on exercise or athletic performance, and they are likely to be retained even in hot environments. However, given the controversies we observed in our thematic analysis, we also speculate that hot environments may modulate the pathways through which caffeine exerts its effects. Therefore, future studies are needed to validate these mechanisms in hot environments, using both human trials and animal models.

### 4.4. Practical Indications and Future Directions

Athletes should exercise caution when using caffeine in high-temperature environments. Moderate caffeine intake may enhance endurance, promote fat oxidation, and improve neural function, but excessive consumption may exacerbate heat stress, leading to elevated heart rate, blood pressure, and body temperature. While proper caffeine use may not significantly increase the risk of dehydration, athletes in hot conditions should monitor their hydration status and implement appropriate rehydration strategies to prevent caffeine’s mild diuretic effect from disrupting fluid balance. Additionally, individual responses to caffeine vary widely, influenced by factors such as genetics, gender, and habitual intake. Therefore, it is advisable to test caffeine’s effects during training to avoid adverse reactions during competition. Caffeine is often consumed alongside other substances, such as carbohydrates or electrolyte drinks, to optimize performance. However, in high-temperature conditions, attention should be given to potential synergistic effects to ensure safe and effective use, avoiding side effects. Caffeine strategies should also align with the specific demands of the event. Athletes may opt to consume caffeine either before competition or in the later stages of endurance activities to delay fatigue and maintain focus. Through careful dosage control, hydration planning, and individualized usage, caffeine may help athletes improve performance in hot environments while mitigating health risks.

To reduce controversy regarding caffeine’s impact on athletic performance in hot conditions, future research should implement several key improvements. First, study designs need to be standardized, including consistent variables such as caffeine dosage, timing of intake, exercise type, and environmental temperature, to ensure comparability across studies. Second, larger sample sizes are needed to increase statistical power and produce more generalizable conclusions. Research should also focus on exploring individual differences, including genetics, gender, age, and habitual caffeine intake, to better understand caffeine’s effects across diverse populations. Furthermore, multi-center collaborative studies are encouraged to integrate data from different regions and populations, enhancing the generalizability and credibility of findings. Studies should also provide detailed descriptions of experimental methods, and data and analysis codes should be made publicly available for verification. In the long term, research should also examine the chronic effects of caffeine, assessing the impact of sustained intake on both performance and health. By integrating perspectives from disciplines such as physiology, nutrition, and psychology, future studies can more comprehensively explore the mechanisms of caffeine’s action, providing clearer evidence to guide athletes in the responsible use of caffeine.

## 5. Conclusions

Caffeine is a common ergogenic aid, but its potential negative effects may be amplified in hot environments, making its impact more complex. In this study, we reviewed the effects of caffeine on exercise and sports in hot environments, primarily using bibliometric methods to describe the current state and development of research in this field. We found that this area has received relatively little attention. Among the six research themes we identified, the results are either highly controversial or remain largely underexplored. Given the current limited collaboration models, we advocate for more scholars to engage in deeper research.

## Figures and Tables

**Table 1:** Basic information of included publications.

Description	Results
Timespan	1994:2024
Sources (Journals, Books, etc.)	19
Documents	35
Annual Growth Rate %	2.34
Document Average Age	11.8
Average citations per doc	16.66
References	657

**Table 2:** Basic charateristics of the included stuides.

Study	Design	Subjects	Nutriention Settings	Caffeine Dosage (mg/kg)	Environment	Exercise
Yu, et al. [46]	Controlled trial	12 college students	Placebo vs. Taurine (TAU) vs. Caffeine (CAF) vs. TAU + CAF		35 °C, 65% RH	Cycling
John, et al. [48]	Controlled trial	12 healthy caffeine-habituated and unacclimatised males	Placebo vs. Caffeine	5	35 °C, 40% RH	Cycling
Naulleau, et al. [23]	Review	52 and 205 endurance-trained individuals	Placebo vs. Caffeine	3 to 9	>27 °C	Endurance Exercise
Cheng, et al. [49]	Controlled trial	12 endurance-trained male collegiate athletes	Placebo vs. Caffeine	6	33 °C, 64% RH	Cycling
Hunt, et al. [50]	Controlled trial	28 habituated nonhabituated individuals (10 females)	Placebo vs. Caffeine	5	30.6 °C, 31% RH	Cycling
Nakamura, et al. [51]		18 male soccer players	Placebo vs. Caffeine	3	32 °C, 70% RH	Cycling
Hanson, et al. [37]	Controlled trial	10 trained endurance runners	Placebo vs. Caffeine	3 and 6	30.6 °C, 50% RH	Running
Bach and Ransone [52]	Controlled trial	21 healthy male subjects	Placebo vs. Caffeine	6	36.37 °C, 59.46% RH	A Maximal Graded Exercise Test (GXT) And Two Endurance Exercise Tests (EET)
Chapman, et al. [53]	Controlled trial	12 healthy subjects (3 females)	Caffeinated Soft Drink vs. Water	n.a.	35 °C, 65% RH	Cycling
Rutherford and Palmer [38]	Controlled trial	6 recreationally active female subjects concurrently taking oral contraceptive steroids	Placebo vs. Caffeine	5.5	29.6 °C, 55.8%	Walking
Beaumont and James [54]	Controlled trial	8 healthy and recreationally active males	Placebo vs. Caffeine	6	30 °C, 50% RH	Cycling
Suvi, et al. [39]	Controlled trial	23 subjects (10 female)	Placebo vs. Caffeine	6	42 °C, 20% RH	Walking
Eaton, et al. [40]	Controlled trial	8 subelite male team sport athletes	Double placebo vs. Essential amino acid (EAC) + placebo vs. Caffeine (CAF) + placebo vs. CAF + EAC	3	30 °C, 20% RH	Running
Beaumont, et al. [55]	Controlled trial	8 recreationally active males	Placebo vs. Caffeine	3 or 1.5 × 2 times	30 °C, 50% RH	Incremental Exercise Test
Pitchford, et al. [56]	Controlled trial	9 well-trained male subjects	Placebo vs. Caffeine	3	35 °C, 25% RH	Cycling
Ely, et al. [57]	Controlled trial	10 not heat-acclimated and not habitual caffeine males	Placebo vs. Caffeine	9	40 °C, 25% RH	Cycling
Ganio, et al. [58]	Controlled trial	11 male cyclists	Placebo vs. Caffeine	3	33 °C, 41% RH	Cycling
Ganio, et al. [59]	Controlled trial	11 male cyclists	Placebo vs. Caffeine	3	12 °C and 33 °C	Cycling
Roelands, et al. [60]	Controlled trial	8 healthy trained male cyclists	Placebo vs. Caffeine	6	30 °C	Cycling
Ping, et al. [41]	Controlled trial	9 heat adapted recreational male runners	Placebo vs. Caffeine	5	31 °C, 70% RH	Running
[61]	Controlled trial	6 males	Caffeinated vs. Non-caffeinated beverages	n.a.	30 °C, 44% RH	n.a.
Del Coso, et al. [62]	Controlled trial	7 endurance-trained heat-acclimated cyclists	Caffeine (CAF) vs. CAF + water vs. CAF + carbohydrate-electrolytes solution	6	36 °C, 29% RH	Cycling
Coso, et al. [63]	Controlled trial	7 endurance-trained cyclists	Caffeine (CAF) vs. CAF + water vs. CAF + carbohydrate-electrolytes solution	6	36 °C, 29% RH	Cycling
Armstrong, et al. [21]	Review	n.a.	n.a.	n.a.	n.a.	n.a.
Roti, et al. [42]	Controlled trial	59 active college-aged males	Placebo vs. Caffeine	0, 3, or 6	37.7 °C, 56.3% RH	Walking
Dias, et al. [43]	Controlled trial	59 active college-aged males	Placebo vs. Caffeine	0, 3, or 6	37.7 °C, 56.3% RH	Walking
Roti, et al. [44]	Controlled trial	59 active college-aged males	Placebo vs. Caffeine	0, 3, or 6	37.7 °C, 56.3% RH	Walking
Stebbins, et al. [64]	Controlled trial	11 caffeine-naive and active men	Placebo vs. Caffeine	6	38 °C	Cycling
Bell, et al. [47]	Controlled trial	10 healthy and non-heat-acclimated males	Placebo vs. Caffeine + ephedrine	5	40 °C, 30% RH	n.a.
Cohen, et al. [45]	Controlled trial	7 endurance-trained competitive road racers (2 famles)	Placebo vs. Caffeine	0, 5, or 9	24–28 °C	Runing
Anderson and Hickey [65]	Controlled trial	8 healthy males	Placebo vs. Caffeine	5	28 °C, 50% RH or 5 °C, 70% RH	Cycling

Note: n.a., not available through the sources or not applicable due to research designs. RH, relative humidity.

**Table 3:** The ten most frequently cited articles.

Paper	DOI	Total Citations	Citation per Year
Armstrong, et al. [21]	10.1097/jes.0b013e3180a02cc1	83	4.61
Coso, et al. [63]	10.1249/MSS.0b013e3181621336	64	3.76
Cohen, et al. [45]	10.1007/BF02425499	53	1.83
Del Coso, et al. [62]	10.1249/MSS.0b013e318184f45e	51	3.19
Roelands, et al. [60]	10.1007/s00421-011-1945-9	50	3.57
Roti, et al. [42]	n.a.	36	1.89
Ganio, et al. [58]	10.1007/s00421-010-1734-x	31	2.21
Stebbins, et al. [64]	10.1046/j.1365-2281.2001.00365.x	25	1.04
Pitchford, et al. [56]	10.1016/j.jsams.2013.07.004	24	2.18
Ely, et al. [57]	10.1123/ijsnem.21.1.65	22	1.57

Note: n.a., not applicable.

## Data Availability

Data are contained within the article.
